# Rising trends of foodborne illnesses in the U.S.: short communication

**DOI:** 10.1097/MS9.0000000000000630

**Published:** 2023-04-11

**Authors:** Abubakar Nazir, Sidhant Ochani, Awais Nazir, Bilquees Fatima, Khushi Ochani, Md. Al Hasibuzzaman, Kaleem Ullah

**Affiliations:** a Department of Medicine, King Edward Medical University, Lahore; b Department of Medicine, Khairpur Medical College, Khairpur Mir’s; c Department of Medicine, Islam Medical College, Sialkot; d Department of Dentistry, Dow University of Health Sciences, Karachi; e Department of Liver Transplantation, Pir Abdul Qadir Shah Jeelani Institute of Medical Sciences, Gambat, Pakistan; f Institute of Nutrition and Food Science, University of Dhaka, Dhaka, Bangladesh

**Keywords:** Foodborne Illness, Infectious Diseases, United States, Salmonella, Listeria monocytogenes, Campylobacter, E. coli

## Abstract

Foodborne illness is caused by the intake of food and water contaminated by different bacteria, viruses, and parasites, as well as poisons or toxins. Approximately 31 different pathogens are documented as causative organisms for causing foodborne illness outbreaks. Climatic changes and varying agricultural practices contribute significantly to the increased incidence of foodborne illness. Foodborne illness can also occur due to the utilization of improperly cooked food. The symptoms of food poisoning may appear sooner or later after contaminated food intake. Symptoms may vary among individuals depending on the disease severity. Despite continuous preventive measures, foodborne illness is still a significant public health threat in the United States. Frequent dining at fast-food restaurants and the use of processed foods present an immense risk of foodborne illness. The food supply in the United States is among the safest in the world, yet we see a surge in foodborne illnesses. People should be encouraged to wash their hands before cooking, and the utensils in which food is being prepared should be kept clean and washed properly before using them. Physicians and other healthcare professionals are facing a host of new challenges in responding to foodborne illnesses. Patients should seek a doctor immediately when they experience symptoms like blood in the stool, hematemesis, prolonged diarrhea for 3 or more days, severe abdominal cramping, and high fever.

Foodborne illness is caused by the intake of food and water contaminated by different bacteria, viruses, and parasites, as well as poisons or toxins^1^. Approximately 31 different pathogens are documented as causative organisms for causing foodborne illness outbreaks. These common pathogens in the U.S. include *Norovirus* (58%), nontyphoidal *Salmonella* species (11%), *Clostridium perfringens* (10%), and *Campylobacter* spp. (9%)^2^. Agrochemicals such as pesticides, plant growth regulators, contaminants such as copper, arsenic, and mercury, several lubricants, and sanitizing agents can also be the cause of this illness^3^. Climatic changes and varying agricultural practices contribute significantly to the increased incidence of foodborne illness^4^,^5^. Foodborne illness can also occur due to the utilization of improperly cooked food. Food poisoning is an illness caused by eating contaminated food; in most cases, the food is contaminated by bacteria, such as *Salmonella* or *Escherichia coli* (*E. coli*), or a virus, such as *Noroviruses*. The symptoms of food poisoning may appear sooner or later after contaminated food intake^5^,^6^. Symptoms may vary among individuals depending on the disease severity. These symptoms include nausea, headache, fatigue, fever, stomach cramps, diarrhea, and vomiting. These symptoms can be life-threatening in certain cases. However, in the majority of patients, the illness is self-limiting, and less often, the patient may need to seek medical advice during a worsening illness. Young children are more prone to foodborne illness, followed by immunocompromised patients, pregnant women, and older adults^6^. Bacteria such as *Campylobacter*, *Salmonella*, and *E. coli* are cultured on agar or other media from stool samples. Parasites can be identified by examining stools under a microscope. Viruses are more difficult to identify, as they are too small to see under a light microscope and are difficult to culture. Viruses can usually have identified by testing stool samples for molecular markers, which are cheap but take time and also by PCR methods which are fast but expensive^4^–^6^.

Despite continuous preventive measures, foodborne illness is still a significant public health threat in the United States. Frequent dining at fast-food restaurants and the use of processed foods that include unhealthy levels of added sugar, sodium, and fat present an immense risk of foodborne illness^7^. The changing trends in food production and distribution, that is the introduction of modern intensive farming to meet the increasing demands, have resulted in the emergence of new pathogens such as *Listeria monocytogenes* 7. Intensive animal farming has also resulted in the emergence of *Salmonella* serotypes. Similarly, the production of biofilms aids the pathogen in colonizing specific food, which causes outbreaks such as the *L. monocytogenes* outbreak that occurred in the U.S. state of Oklahoma^7^. In a food processing plant, a contaminated food ingredient can result in the contamination of several batches of the final product. The wide distribution of such contaminated products can result in an epidemic, and previously *Salmonella typhimurium* outbreaks were also linked to the use of peanut butter^8^. Import of contaminated food with disease-causing pathogens may be another reason for the increased incidence of foodborne illness^9^. Unsafe agricultural practices, such as the use of organic waste as a fertilizer, further aggravate the spread of pathogens^10^. Antibiotic resistance has enabled pathogens to survive varying environmental conditions. Physiological changes resulted in the occurrence of new multidrug-resistant pathogens. For example, cases of multidrug-resistant strains of *Salmonella* have been recorded, which represent a significant threat to food safety^11^.

The federal government estimates that there are 48 million cases reported every year, which is equal to one in six Americans, which results in 128 000 hospitalizations and 3000 deaths annually^11^. Outbreaks of foodborne diseases occur more in Southern parts of the USA due to low socioeconomic status. A significant surge in *Campylobacter* infection and *Shigella* rates due to eating contaminated food has been observed since 2015 in the U.S.^4^,^5^. According to the CDC, 65 outbreaks were caused by *Salmonella*, *E. coli* [Shiga toxin-producing *E. coli* (STEC)], and *L. monocytogenes* from 2017to 2020 out of which 52 were associated with contaminated foods and 7659 people were affected with an average of 1900 cases per year, 2044 were hospitalized, and 41 died then^12^. As of September 2022, the total number of cases of *Salmonella* species, *L. monocytogenes*, and *E. coli* are 144, 20, and 97, respectively, indicating an 8% rise in overall foodborne cases in just one month compared to the average annual incidence^13^.

The food supply in the United States is among the safest in the world, yet we see a surge in foodborne illnesses. People should be encouraged to wash their hands before cooking, and the utensils in which food is being prepared should be kept clean and washed properly before using them. Awareness regarding confirming the expiry dates of food items before use is needed. People should be educated regarding fecal–oral transmission. People should be advised to avoid raw, undercooked poultry, meat, and eggs as they act as a source of *Campylobacter*, *Salmonella*, and *E. coli* infection. The general population should be facilitated with water filters in rural areas by the government. Emphasis should be made on drinking boiled water and milk to avoid the risk of spreading *Listeria*. Also, reheated food intake should be discouraged.

Physicians and other healthcare professionals are facing a host of new challenges in responding to foodborne illnesses. Patients should seek a doctor immediately when they experience symptoms like blood in the stool, hematemesis, prolonged diarrhea for 3 or more days, severe abdominal cramping, and high fever. Antibiotics should be considered only on practitioner advice. Healthcare providers should direct educational efforts toward all people with diarrhea, particularly people with primary and secondary immune deficiencies, pregnant women, parents of young children, and the elderly, as they have an increased risk of complications from diarrheal disease.

## Ethical approval

Not applicable.

## Consent

Not applicable.

## Sources of funding

The author(s) received no financial support for the research, authorship, and/or publication of this article.

## Author contribution

A.N., S.O., A.N., B.F., and K.O.: writing the manuscript; S.O., K.O., M.A.H., and K.U.: review editing, formatting, and referencing. The literature review was done by all the authors.

## Conflicts of interest disclosure

The author(s) declared no potential conflicts of interest concerning the research, authorship, and/or publication of this article.

## Provenance and peer review

Not commissioned, externally peer-reviewed.
